# Extracellular vesicles derived from microRNA-150-5p-overexpressing mesenchymal stem cells protect rat hearts against ischemia/reperfusion

**DOI:** 10.18632/aging.102792

**Published:** 2020-07-13

**Authors:** Hesheng Ou, Hongli Teng, Yuwang Qin, Xuelan Luo, Peng Yang, Wenyu Zhang, Wei Chen, Dongning Lv, Huamin Tang

**Affiliations:** 1 Section of Science and Technology, Guangxi International Zhuang Medicine Hospital Affiliated to Guangxi University of Chinese Medicine, Nanning 530201, P.R. China; 2 Research and Development Center of Zhuang and Yao Medicine, Guangxi International Zhuang Medicine Hospital Affiliated to Guangxi University of Chinese Medicine, Nanning 530201, P.R. China; 3 Section of Drug and Equipment, The Central Hospital of China Railway 12th Bureau Group Co. Ltd, Taiyuan 030053, P.R. China; 4 Department of Pharmacy, Jinan Maternity and Child Care Hospital, Jinan 250001, P.R. China; 5 Section of Nephropathy, Pulmonary Diseases and Endocrinology, Guangxi International Zhuang Medicine Hospital Affiliated to Guangxi University of Chinese Medicine, Nanning 530201, P.R. China; 6 Emergency First Aid Linkage Center, Guangxi International Zhuang Medicine Hospital Affiliated to Guangxi University of Chinese Medicine, Nanning 530201, P.R. China

**Keywords:** ischemia/reperfusion, myocardial remodeling, mesenchymal stem cells, extracellular vesicles, microRNA-150-5p

## Abstract

An intriguing area of research has demonstrated the ability of extracellular vesicles (EVs) as biological vehicles for microRNAs (miRNAs) transfer. Mesenchymal stem cells (MSCs) produce large amounts of EVs. Rat models of ischemia/reperfusion (I/R) were established to explore the expression profile of thioredoxin-interacting protein (TXNIP), which was then knocked-down to investigate its effects on myocardial remodeling, followed by detection on myocardial infarction size (MIS), myocardial collagen volume fraction (CVF) and cardiomyocyte apoptosis. MSCs-derived EVs carrying miR-150-5p were cultured with neonatal cardiomyocytes under hypoxia/hypoglycemia condition for *in vitro* exploration and intramyocardially injected into I/R rats for *in vivo* exploration. I/R-induced rats presented higher TXNIP levels and lower miR-150-5p levels, along with increased cardiomyocyte apoptosis. miR-150-5p in MSCs was transferred through EVs to cardiomyocytes, leading to suppressed myocardial remodeling, as reflected by smaller MIS and CVF and suppressed cardiomyocyte apoptosis. I/R-treated rats injected with MSCs-derived EVs containing miR-150-5p showed a reduction in myocardial remodeling associated with the downregulation of TXNIP, which may be clinically applicable for treatment of I/R.

## INTRODUCTION

Myocardial infarction (MI) is an injury of heart muscle caused by insufficient supply of blood [1]. In 2015, roughly 16 million people suffered myocardial infarctions throughout the world and this disease causes heavy healthcare expenditure for patients [2, 3]. Multiple means attempting to restore blood flow should be applied immediately after MI occurs. One of the effective methods is blood reperfusion. However, it always leads to cardiomyocyte death unexpectedly and loss of regular physiological function further, which is referred as myocardial ischemia-reperfusion (I/R) injury [1]. The pathological causes of myocardial I/R injury are complicated, but are most commonly attributed to damaged homeostasis of oxygen demand and an imbalance in ion concentration. During reperfusion, destiny of cardiomyocytes is demonstrated to be determined by mitochondrial permeabilization. Severe break of mitochondria causes death of cardiomyocytes by inducing apoptosis and necrosis [4]. A quick recovery of intracellular pH can also be found during reperfusion, which can cause a high rate of 2Na^+^/Ca2^+^ exchange and overload of Ca2^+^ cation [5]. In addition, supplement of oxygen by reperfusion can elevate oxidation of fatty acid and anaerobic glycolysis, causing inadequate energy acquisition by cardiomyocytes [6]. Therefore, any methods that balance metabolism in cardiomyocytes should be investigated.

Reperfusion is always accompanied by altered expression of critical genes. For example, some protein kinases in the RISK pathway are activated during reperfusion to prevent lethal injury of cardiomyocytes, acting as cardiac protectors [7]. Reactive oxygen species (ROS) are reported to be associated with reperfusion injury [8]. Genes in ROS signaling pathway are believed to regulate myocardial I/R injury. TXNIP is a thioredoxin-interacting protein and has been reported to induce arrest of cell cycle, which acts as a tumor suppressor [9]. However, the molecular mechanism by which TXNIP affects myocardial I/R injury is unclear.

Extracellular vesicles (EVs) are lipid vesicles outside cells carrying functional RNAs and proteins to other cells [10]. EVs have been reported to play important roles in immune system and tumor progression [11, 12]. The first report to suggest a role for EVs in myocardial I/R injury was published in 2010. Lai et al. showed that EVs from mesenchymal stem cells (MSCs) could significantly reduce the damages caused by myocardial I/R injury [13]. The mechanism that MSCs-derived EVs ameliorated myocardial I/R injury conditions was partly uncovered three years later which was associated with the PI3K/Akt signaling pathway [14]. EVs are known to transfer microRNAs (miRNAs) from one cell to another as a means of communication. miRNAs, endogenously produced in eukaryotic cells, induce gene-specific silencing by elevated degradation or translational inhibition of mRNA through incomplete complementary sequence binding [15, 16]. Most miRNAs are intergenic, and located in noncoding regions between genes and transcribed by unidentified promoters [17]. How miRNAs carried by EVs regulate TXNIP and further affect myocardial I/R injury damage is less studied. Therefore, in the present study, we reported miR-150-5p carried by MSCs-derived EVs reduced myocardial remodeling in a rat model with I/R, providing a promising treatment of myocardial I/R injury via delivery of EVs secreted from MSCs.

## RESULTS

### TXNIP is upregulated in I/R-induced rats

**Figure 1:**
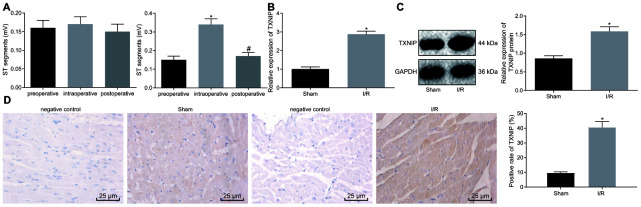
**TXNIP is highly expressed in the rat model of I/R.** (**A**) The preoperative, intraoperative and postoperative ST segments (mV) in sham-operated rats (n = 12) (left) and in rats with I/R (n = 72) (right); * *p* < 0.05 *vs.* the preoperative ST segment; # *p* < 0.05 *vs.* the intraoperative ST segment. (**B**) TXNIP expression in myocardium determined by RT-qPCR; * *p* < 0.05 *vs.* the sham group (sham-operated rats). (**C**) TXNIP protein expression in myocardium normalized to GAPDH determined by Western blot analysis; * *p* < 0.05 *vs.* the sham group (sham-operated rats). (**D**) The positive expression of TXNIP in myocardium identified by immunohistochemistry (400 ×); * *p* < 0.05 *vs.* the sham group (sham-operated rats). Measurement data were presented as mean ± standard deviation. Comparison between two groups was analyzed by unpaired *t*-test. Comparison among multiple groups was analyzed by one-way analysis of variance, followed by Tukey’s post hoc test. n = 12.

First, rat models of I/R were induced for exploration purpose in vivo. Results revealed no significant differences regarding preoperative, intraoperative and postoperative ST segment of ECG in sham-operated rats. Rats with I/R exhibited a significantly higher ST segment after ligation than before, which was reduced after reperfusion, indicating that the conduction of vessel occlusion exerted no obvious effects on cardiac electrophysiology of rats. Significant difference in ST segment was observed after ligation of LAD in comparison to ST segment during ligation. Following reperfusion, cardiac electrophysiology was recovered, suggesting the successful establishment of the I/R rat model (Figure 1A and Supplementary Figure 1). All procedures were performed on surviving rats without malignant arrhythmia. The survival rate of sham-operated rats was 100% while that of I/R-induced rats was 94.44% on the 28^th^ day post modeling. Meanwhile, to investigate the effects of TXNIP on myocardial injury of rats with I/R, the expression profile of TXNIP was determined in the myocardium on the ischemic border area. As shown in Figure 1B, 1C, TXNIP expression significantly increased in rats with I/R compared to sham-operated rats (p < 0.05). The immunohistochemistry results further confirmed that positive expression of TXNIP in the cytoplasm was significantly higher in rats with I/R that in sham-operated rats (Figure 1D). The aforementioned findings demonstrate that TXNIP is highly expressed in rats with I/R.

### TXNIP knockdown inhibits the myocardial remodeling in I/R-induced rats

**Figure 2:**
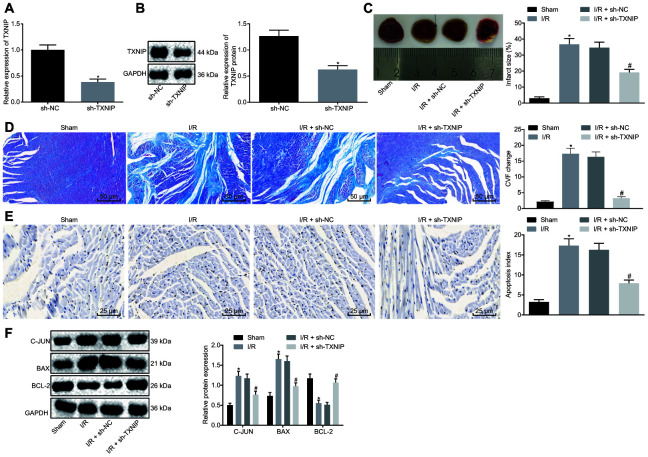
**TXNIP knockdown impedes myocardial remodeling of rats with I/R.** (**A**) TXNIP expression in myocardium determined by RT-qPCR. (**B**) TXNIP protein expression in myocardium normalized to GAPDH determined by Western blot analysis. (**C**) The myocardial infarct size detected by TTC staining. (**D**) The myocardial collagen detected by Masson's staining (200 ×). (**E**) The cardiomyocyte apoptosis detected by TUNEL staining (400 ×). (**F**) The protein expression of apoptosis-related factors (c-Jun, Bax and Bcl-2) normalized to GAPDH determined by Western blot analysis. * *p* < 0.05 *vs.* the sham group (sham-operated rats); # *p* < 0.05 *vs.* the I/R + sh-NC group (I/R rats treated with sh-NC). Measurement data were presented as mean ± standard deviation. Comparison between two groups was analyzed by non-paired *t*-test. n = 12.

Given the elevated expression of TXNIP, TXNIP was knocked down to investigate its role in I/R injury. The knockdown efficiency of TXNIP was assessed by Reverse transcription quantitative polymerase chain reaction (RT-qPCR) and Western blot analysis. Figure 2A, 2B revealed that mRNA and protein expression of TXNIP significantly declined after knockdown. Assessment of cardiac functions (Table 1) revealed that rats with I/R had significantly higher levels of LVEDV, LVEDD, LVESV and LVESD along with lower levels of LVEEF and LVEFS. Opposite changing tendency was detected in I/R rats with TXNIP knockdown when compared with I/R rats with sh-NC. Subsequently, TTC and Masson's staining assays were used to evaluate MIS and CVF, respectively, both of which increased in I/R rats and decreased in the presence of TXNIP knockdown (Figure 2C, 2D). TUNEL staining was then performed to detect cardiomyocyte apoptosis, followed by Western blot analysis for quantification of apoptosis-related factors (c-Jun, Bax and Bcl-2). It was found that, cardiomyocyte apoptosis was promoted in I/R rats accompanied by higher levels of c-Jun and Bax along with lower Bcl-2 level but suppressed following treatment of TXNIP knockdown in parallel with lower levels of c-Jun and Bax along with higher Bcl-2 level (Figure 2E, 2F). These findings indicate that myocardial remodeling in rats with I/R can be impeded by TXNIP knockdown.

**Table 1:** Echocardiograph results of LVEDV, LVEDD, LVESV, LVESD, LVEEF and LVEFS by echocardiography.

**Group**	**LVEDV (μL)**	**LVEDD (mm)**	**LVESV (μL)**	**LVESD (mm)**	**LVEEF (%)**	**LVEFS (%)**
Sham	216.12 ± 18.37	5.47 ± 0.23	85.45 ± 7.46	3.12 ± 0.28	60.29 ± 4.29	42.94 ± 4.67
I/R	400.23 ± 32.38*	11.49 ± 1.11*	287.46 ± 25.38*	10.23 ± 1.03*	28.18 ± 2.68*	10.98 ± 1.10*
I/R + sh-NC	401.83 ± 24.18	12.35 ± 1.05	284.88 ± 22.35	11.16 ± 0.95	29.12 ± 3.09	9.61 ± 0.83
I/R + sh-TXNIP	285.49 ± 24.39#	8.18 ± 0.47#	165.42 ± 17.56#	5.84 ± 0.47#	41.98 ± 4.79#	28.65 ± 3.04#
I/R + EV^agomir-NC^	380.48 ± 26.90	11.58 ± 0.34	259.96 ± 29.23	10.19 ± 0.41	31.84 ± 3.09	12.02 ± 1.49
I/R + EV^miR-150-5p-agomir^	274.35 ± 19.78&	7.95 ± 0.55&	161.13 ± 15.83&	6.00 ± 0.54&	41.34 ± 2.09&	24.50 ± 2.65&
I/R + EV^miR-150-5p-agomir^ + oe-TXNIP	416.01 ± 19.97$	11.59 ± 0.32$	301.12 ± 22.14$	10.20 ± 0.39$	27.63 ± 3.61$	11.99 ± 1.49$

Note: * *p* < 0.05 *vs.* the sham group (sham-operated rats); # *p* < 0.05 *vs.* the I/R + sh-NC group (I/R rats treated with sh-NC); & *p* < 0.05 *vs.* the I/R + EV^agomir-NC^ group (I/R rats treated with EV^agomir-NC^); $ *p* < 0.05 *vs.* the I/R + EV^miR-150-5p-agomir^ group (I/R rats treated with EV^miR-150-5p-agomir^). The measurement data were expressed as mean ± standard deviation. Comparison among multiple groups was conducted using one-way analysis of variance, followed by Tukey’s post hoc test. I/R, ischemia/reperfusion; NC, negative control; TXNIP, thioredoxin-interacting protein; miR, microRNA; LVEDV, left ventricular end-diastolic volume; LVEDD, left ventricular end-diastolic dimension; LVESV, left ventricular end-systolic volume; LVESD, left ventricular end-systolic dimension; LVEEF, left ventricular ejection fraction; LVEFS, left ventricular fractional shortening.

### MiR-150-5p targets and negatively regulates TXNIP

**Figure 3:**
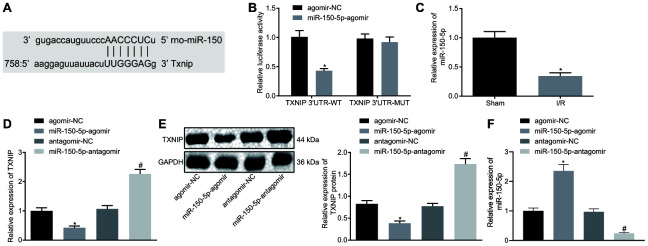
**TXNIP is the target of miR-150-5p.** (**A**) The binding sites between miR-150-5p and TXNIP as predicted by microrna.org. (**B**) The relative luciferase activity determined by dual-luciferase reporter gene assay. (**C**) The miR-150-5p expression in myocardium determined by RT-qPCR, normalized to U6; * *p* < 0.05 *vs.* the sham group (sham-operated rats); n =12. (**D**) The mRNA expression of TXNIP in myocardium determined by RT-qPCR, normalized to GAPDH. (**E**) The protein expression of TXNIP in myocardium normalized to GAPDH determined by Western blot analysis. (**F**) The expression of miR-150-5p in cardiomyocytes in response to miR-150-5p-agomir and miR-150-5p-antagomir determined by RT-qPCR. * *p* < 0.05 *vs.* the agomir-NC group (I/R rats treated with agomir-NC); # *p* < 0.05 *vs.* the antagomir-NC group (I/R rats treated with antagomir-NC). Measurement data were presented as mean ± standard deviation. Comparison between two groups was analyzed by unpaired *t*-test. The cell experiment was repeated 3 times independently.

Prediction software at microrna.org identified binding sites between miR-150-5p and TXNIP (Figure 3A). The binding between them was confirmed using the dual-luciferase reporter gene assay. The luciferase activity of TXNIP 3’UTR-WT was significantly inhibited by miR-150-5p-agomir but that of TXNIP 3’UTR-MUT remained almost unchanged (Figure 3B). RT-qPCR revealed that miR-150-5p expression was significantly lower in I/R rats than sham-operated rats (Figure 3C). Next, miR-150-5p in neonatal cardiomyocytes was overexpressed and inhibited to figure out the relation between miR-150-5p and TXNIP by means of RT-qPCR and Western blot analysis. Results showed that mRNA and protein expression of TXNIP remarkable decreased following miR-150-5p overexpression but increased following miR-150-5p inhibition (Figure 3D, 3E). Besides, miR-150-5p expression increased in response to delivery of miR-150-5p-agomir and decreased in response to delivery of miR-150-5p-antagomir (Figure 3F). Taken together, miR-150-5p targets TXNIP and negatively regulates its expression.

### MSCs-derived EVs carrying MiR-150-5p inhibit cardiomyocyte apoptosis

**Figure 4:**
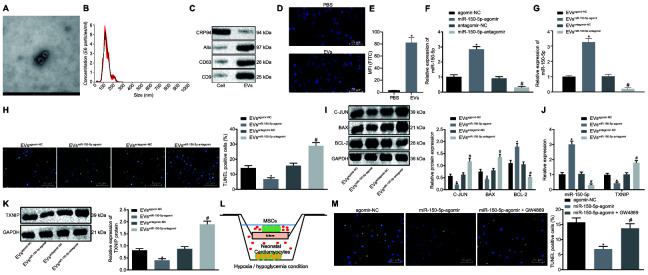
**Cardiomyocyte apoptosis is suppressed by MSCs-derived EVs carrying miR-150-5p.** (**A**) The images captured under transmission electron microscopy. (**B**) EV size measured by nanoparticle tracking analysis. (**C**) The expression of EV markers (CD9, CD63, Alix and GRP94) detected by Western blot analysis. (**D**) EV internalization detected by immunohistochemistry (400 ×). (**E**) PKH67-staining (FITC wavelength) in neonatal cardiomyocytes detected by flow cytometric analysis. (**F**) The expression of miR-150-5p in MSCs determined by RT-qPCR. (**G**) The expression of miR-150-5p in EVs determined by RT-qPCR. (**H**) neonatal cardiomyocyte apoptosis detected by TUNEL staining (200 ×). (**I**) The protein expression of apoptosis-related factors (c-Jun, Bax and Bcl-2) normalized to GAPDH determined by Western blot analysis. (**J**) The expression of miR-150-5p and TXNIP in neonatal cardiomyocytes determined by RT-qPCR. (**K**) TXNIP expression in neonatal cardiomyocytes normalized to GAPDH determined by Western blot analysis. (**L**) Transwell co-culture system under hypoxia/hypoglycemia condition. (**M**) Neonatal cardiomyocyte apoptosis detected by TUNEL staining (200 ×); * *p* < 0.05 *vs.* the agomir-NC group (cells treated with agomir-NC); # *p* < 0.05 *vs.* the miR-150-5p-agomir group (cells treated with miR-150-5p-agomir). * *p* < 0.05 *vs.* the agomir-NC group (cells treated with agomir-NC) or the EV^agomir-NC^ group (cells treated with EV^agomir-NC^); # *p* < 0.05 *vs.* the antagomir-NC group (cells treated with antagomir-NC) or the EV^antagomir-NC^ group (cells treated with EV^antagomir-NC^). Measurement data were presented as mean ± standard deviation. Comparison among multiple groups was analyzed by one-way analysis of variance, followed by Tukey’s post hoc test. The cell experiment was repeated 3 times independently.

According to results of high resolution Transmission electron microscopy (TEM), MSCs-derived EVs presented in round shape with double membrane (Figure 4A) and EVs had a dimension range from 30 to 200 nm as revealed by nanoparticle tracking analysis (Figure 4B). Western blot analysis results confirmed the high expression of EVs-positive markers (CD9, CD63 and Alix) and poor expression of negative marker GRP94 (Figure 4C). Then, EVs were labeled by PKH67 (green fluorescence) and co-cultured with neonatal cardiomyocytes. Immunocytochemistry results demonstrated that EVs could be internalized by neonatal cardiomyocytes since PKH67 (green fluorescence) was detected inside neonatal cardiomyocytes (Figure 4D). Similar results were observed through flow cytometry that much more PKH67 (green fluorescence) was detected inside neonatal cardiomyocytes following co-culture with PKH67-labeled EVs when compared with co-culture with PBS (Figure 4E). To evaluate whether MSCs-derived EVs could function as a transfer system of miR-150-5p, we isolated EVs from MSCs after treatment of miR-150-5p overexpression and inhibition (Figure 4F). RT-qPCR showed that miR-150-5p expression increased significantly in EVs from MSCs treated with miR-150-5p-agomir but decreased significantly in EVs from MSCs treated with miR-150-5p-antagomir in comparison to their corresponding NC (Figure 4G). Subsequently, EVs were further co-cultured with neonatal cardiomyocytes under exposure to hypoxia/hypoglycemia. TUNEL staining and Western blot analysis of cardiomyocyte apoptosis revealed that miR-150-5p-agomir-treated EVs exhibited a significant reduction in TUNEL-positive cells as well as a decrease in c-Jun and Bax protein levels while Bcl-2 protein was increased. Treatment with miR-150-5p-antagomir had the opposite results (Figure 4H, 4I). Next, expression of miR-150-5p and TXNIP was determined in neonatal cardiomyocytes and results revealed increased miR-150-5p expression and decreased mRNA and protein levels of TXNIP in the presence of miR-150-5p-agomir while opposite changing tendency was detected in the presence of miR-150-5p-antagomir (Figure 4J, 4K). Then, we directly co-cultured MSCs expressing upregulated miR-150-5p with neonatal cardiomyocytes using a Transwell system (Corning, NY, USA) (Figure 4L). Besides, TUNEL staining was performed for cell apoptosis evaluation that delivery of miR-150-5p-agomir led to decreased apoptosis rate of neonatal cardiomyocytes while the addition of 10 μM GW4869 (EV blocking agent, Sigma-Aldrich, St. Louis, MO, USA) rescued the neonatal cardiomyocyte apoptosis (Figure 4M). These findings shed light on the suppressive effects of MSCs-derived EVs carrying miR-150-5p on cardiomyocyte apoptosis.

### EVs-loaded MiR-150-5p inhibits myocardial remodeling following I/R by regulating TXNIP

**Figure 5:**
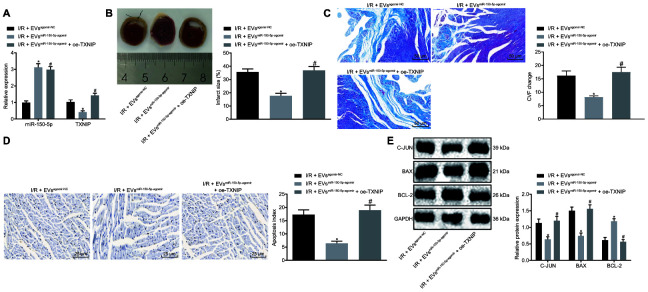
**The EVs-loaded with miR-150-5p/TXNIP mediates myocardial remodeling after I/R.** (**A**) The transduction efficiency detected by RT-qPCR. (**B**) The myocardial infarct size detected by TTC staining. (**C**) The myocardial collagen detected by Masson's staining (200 ×). (**D**) The cardiomyocyte apoptosis detected by TUNEL staining (400 ×). (**E**) The protein expression of apoptosis-related factors (c-Jun, Bax and Bcl-2) normalized to GAPDH determined by Western blot analysis. * *p* < 0.05 *vs.* the I/R + EV^agomir-NC^ group (I/R rats treated with EV^agomir-NC^); # *p* < 0.05 *vs.* the I/R + EV^miR-150-5p-agomir^ group (I/R rats treated with EV ^miR-150-5p-agomir^). Measurement data were presented as mean ± standard deviation. Comparison among multiple groups was analyzed by one-way analysis of variance, followed by Tukey’s post hoc test. n = 12.

Next, we explored the mechanism by which miR-150-5p and TXNIP regulate myocardial remodeling after I/R injury. RT-qPCR was used to detect the transduction efficiency of I/R rats with results shown in Figure 5A, signaling the initiation of following analysis. Cardiac function assessment (Table 1) demonstrated that LVEDV, LVEDD, LVESV and LVESD were significantly decreased while LVEEF and LVEFS were increased in I/R rats following treatment of EV^miR-150-5p-agomir^. However, the addition of oe-TXNIP rescued the effects induced by EV^miR-150-5p-agomir^. TTC and Masson's staining assays showed that oe-TXNIP also reversed the inhibitory effects of EV^miR-150-5p-agomir^ on MIS and CVF (Figure 5B, 5C). As for cardiomyocyte apoptosis detection using TUNEL staining and Western blot analysis, EV^miR-150-5p-agomir^ treatment lowered the apoptosis rate and decreased c-Jun and Bax protein levels while it increased Bcl-2 protein level. Expectedly, such alternations could be rescued by oe-TXNIP (Figure 5D, 5E). The above-mentioned results suggest that EVs-loaded miR-150-5p regulates TXNIP to further inhibit myocardial remodeling after I/R.

## DISCUSSION

Animal studies have showed that myocardial I/R injury can account for up to 50% of the final size of myocardial infarction region [18]. Multiple cardioprotective strategies have been applied to reduce the damage from myocardial I/R injury including surgical operations such as inflation and deflation of coronary-angioplasty balloon immediately after stent deployment, usage of peptides or kinase inhibitors [5]. In the study, we investigated the effect of EVs combined with upstream miRNA of TXNIP in a rat model of I/R. We found that miR-150-5p specifically targeted TXNIP and MSCs-derived EVs carrying miR-150-5p could inhibit cardiomyocyte apoptosis *in vitro* as well as suppressed myocardial remodeling *in vivo* after myocardial I/R injury.

We found TXNIP was overexpressed in myocardial tissue of I/R rat models. TXNIP is a negative regulator of Trx1. Overexpression of TXNIP increases the sensitivity of cardiomyocytes to oxidative stresses [19]. TXNIP was also upregulated in the peripheral leukocytes of patients with myocardial infarction or patients with coronary atherosclerotic heart disease, which suggests that circulating leucocytes also responded to the oxidative stress in the blood and participated in the pathogenesis of heart disease [20, 21]. Therefore, TXNIP might be considered as a biomarker in myocardial I/R injury considering its role on oxidative stresses.

Furthermore, we found that miR-150-5p carried by MSCs-derived EVs could reduce myocardial remodeling both *in vitro* and *in vivo* via downregulation of TXNIP. miR-150 has been reported as a novel marker for myocardial infarction. Studies show that some miRNAs correlate to process of proliferation, differentiation and apoptosis of cells as well as the development of various human diseases including cardiovascular diseases [22, 23]. For example, miR-150 was downregulated in patients with left ventricular remodeling compared with negative group [24]. In a mouse model of acute myocardial infarction, miR-150 reduced damage to the heart by inhibiting the accumulation of monocytes [25]. Also, in ischemic injury models, miR-150 could significantly reduce myocardial death, thus protecting mouse hearts from ischemia [26]. These studies indicate that miR-150 has a protective role against heart diseases; however, few studies directly investigate the role of miR-150 in myocardial I/R injury. One study reported that overexpression of miR-150 reduced apoptosis of human cardiomyocytes induced by hypoxia via targeting another protein, GRP94 [27]. Nevertheless, it seemed contradictory that miR-150 was upregulated in H_2_O_2_ treated cardiomyocytes. Overexpression of miR-150 increases cardiac cell apoptosis and death induced by H_2_O_2_ by downregulating c-myc expression [28]. The opposite results caused by miR-150 in ischemia reperfusion and ROS of H2O2 might be the distinct targets by miR-150 and the different subsequent signaling pathway downstream these targets.

MSCs are multipotent stem cells, which can differentiate into multiple cell types, such as muscle cells, fat cells, bone cells and cartilage cells [29]. Because of their differentiation capacity and immunomodulatory effects, MSCs have been studied in autoimmune disease and other diseases [30]. EVs secreted by MSCs serve as an efficient delivery system for the transport of target molecules. Delivery systems of MSCs-derived EVs exhibit three advantages. First, MSCs especially bone marrow MSCs are easily to be obtained and cultured in artificial medium and can be differentiated into certain cell types under proper conditions. Second, EVs are lipid structures containing membrane proteins with little immunogenicity, which means they can be widely used in many types of cells. Third, EVs can carry abundant biomolecules, including miRNAs, and transport them into target cells by membrane fusion. Many studies applying MSCs/EVs in heart diseases have obtained good results. For instance, transplantation of MSCs-released EVs carrying miR-125b significantly reduced autophagic flux in mouse myocardial infarction model [31]. EVs-loaded miR-21a-5p secreted by MSCs suppressed proapoptotic genes including PDCD4, PTEN, Peli1 and FasL and played a cardioprotective role in myocardial cells [32]. Likewise, EVs carrying miR-93 inhibited hypoxia-induced autophagy and the expression of inflammatory cytokines by specifically downregulating Atg7 and TLR4, respectively. These studies highlight the promising applications of MSCs-derived EVs in the treatment of heart disease in the future.

**Figure 6:**
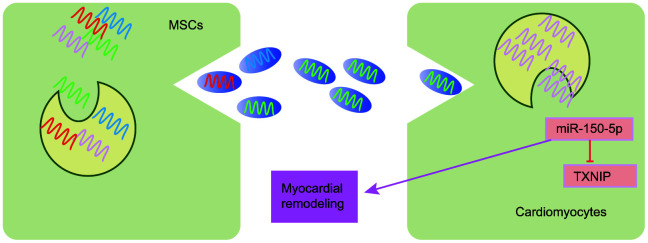
**The schematic diagram depicts molecular basis underlying EVs derived from MSCs on I/R by transferring miR-150-5p.** MSCs can secrete EVs to transfer miR-150-5p into cardiomyocytes, which further internalize EVs to release miR-150-5p and miR-150-5p protects against I/R by downregulating TXNIP and inhibiting myocardial remodeling.

Conclusively, we found significantly highly expressed TXNIP and poorly expressed miR-150-5p in heart tissues of myocardial I/R injury. Either addition of EVs extracted from MSCs in the medium culturing cardiomyocytes or coculture of MSCs with cardiomyocytes efficiently reduced cellular apoptosis *in vitro*. In rat models with I/R, injection of EVs carrying miR-150-5p or MSCs overexpressing miR-150-5p both reduced myocardial I/R injury via downregulation of TXNIP (Figure 6). However, the exact molecular mechanisms by miR-150-5p/TXNIP to improve myocardial I/R injury need further investigations. Otherwise, more trials have to be carried out in animal models before human tests.

## MATERIALS AND METHODS

### Study subjects

A total of 90 healthy male Sprague Dawley (SD) rats (age: 7 – 8 weeks, weight: 250 – 280 g) (Guangdong Medical Laboratory Animal Center, Foshan, Guangdong, China) were housed at 20 - 25°C in a constantly humidified atmosphere under a 12-h light/dark cycle with free access to water and food. The animal treatment was performed in strict accordance with the recommendations in the Guide for the Care and Use of Laboratory Animals of Guangxi International Zhuang Medical Hospital Affiliated to Guangxi National University of Traditional Chinese Medicine.

MSCs were isolated from healthy 8-week SD rats with reference to the methods reported previously [33, 34] and cultured in high glucose Dulbecco's Modified Eagle Medium (DMEM) supplemented with 10% fetal bovine serum (FBS) and 1% antibiotics (streptomycin and penicillin).

Neonatal cardiomyocytes were isolated from 2-day newborn rats using the Neonatal Cardiomyocytes Isolation kit (Worthington Biochemical Corp., Lakewood, NJ, USA) and cultured in DMEM (Hyclone, Logan, Utah, USA) containing 10% FBS.

### Establishment of rat models with I/R

In total, 72 SD rats that had undergone 72-h adaptation period were selected for I/R modeling. Prior to the operation, all rats were anesthetized with 10% pentobarbital sodium (90 mg/kg, wS20060401, Shanghai Westang Biotechnology Co., Ltd., Shanghai, China). The four limbs of rats were connected to the monitoring electrodes of an electrocardiogram (ECG) (BeneHearth R3, Mindray, Wuhan, China) with ventilation coming from animal respirator (R407, RWd Biotech Co., Ltd., Shenzhen, China) (gas source: room air, ventilator frequency: 60 breaths/min, tidal volume: 13 - 15 mL/kg). The heart was exposed by left thoracotomy through the 4^th^ intercostal space, and the left anterior descending (LAD) coronary artery was ligated using 6/0 atraumatic suture. ST-segment elevation detected by ECG monitoring suggested the successful occlusion of vessels. Local ischemia was confirmed by observing the discoloration of the distal occlusive myocardium with naked eyes under the anatomical microscope (NIKON, Tokyo, Japan). Then, 30 min post occlusion, the ligation was released and the tissues were re-perfused for 24 h. Next, the rats were kept overnight in a cage at 37°C [35, 36].

### Animal grouping

A total of 12 sham-operated rats served as the sham group. After thoracotomy, those rats were ligated using 6/0 atraumatic suture in the LAD coronary artery without vessel occlusion. The 72 rats that received operation for I/R were randomly assigned into 6 groups (12 rats each group) as follows: 1) the I/R group; 2) the I/R + sh-negative control (sh-NC) group (rats intramyocardially injected with 10^8^ U sh-NC 10 min before perfusion, an average of 5 injections, 2 μL per injection, 10 μL in total); 3) the I/R + sh-TXNIP group (rats intramyocardially injected with 10^8^ U sh-TXNIP 10 min before perfusion, 5 injections averagely, 2 μL per injection, 10 μL in total); 4) the I/R + EV^miR-150-5p-agomir^ group (rats intramyocardially injected with EVs containing miR-150-5p-agomir 10 min before perfusion, 5 injections averagely, 10 μL per injection, 50 μL in total, 5.8 × 10^12^ particles); 5) the I/R + EV^agomir-NC^ group (rats intramyocardially injected with EVs containing agomir-NC 10 min before perfusion, 5 injections averagely, 10 μL per injection, 50 μL in total, 5.8 × 10^12^ particles); 6) the I/R + EV^miR-150-5p-agomir^ + oe-TXNIP group (rats intramyocardially injected with 10^8^ U oe-TXNIP and EVs containing miR-150-5p-agomir 10 min before perfusion, oe-TXNIP: 5 injections averagely, 2 μL per injection, 10 μL in total; EVs: 5 injections averagely, 10 μL per injection, 50 μL in total, 5.8 × 10^12^ particles). All lentivirus vectors were purchased from Yunnan Ruibao Biological Co., Ltd., China. In total, 4 rats died with a mortality rate of 5.6% during the operative procedure, while the remaining 6 rats were used as alternates. After 24 h of reperfusion, rats were anesthetized with 1% sodium pentobarbital (40 mg/kg) and euthanized to obtain myocardial tissues.

### Cell grouping and transduction

MSCs at passage 2 – 4 and neonatal cardiomyocytes were transduced with agomir-NC (50 nM), miR-150-5p-agomir (50 nM), antagomir-NC (100 nM) and miR-150-5p-antagomir (100 nM), respectively, in accordance with the manual of Lipofectamine 2000 (Invitrogen, Carlsbad, CA, USA). The culture medium was renewed by complete medium after culture at 37°C with 5% CO_2_ for 6 – 8 h. The subsequent experiments proceeded after culture for another 24 – 48 h. MSCs after transduction were adopted for further EV isolation.

Neonatal cardiomyocytes were plated in a 6-well plate at a density of 2 × 10^4^ cells/well, added with 2 mL DMEM and cultured in an incubator under constant temperature with CO_2_ for 24 h to make cells grow adhere to the plate. Then, cells were transduced with 1000 ng/mL EV^agomir-NC^, EV^miR-150-5p-agomir^, EV^antagomir-NC^ and EV^miR-150-5p-antagomir^.

### Establishment of a cell model

The glucose-free medium was exposed to hypoxic condition (95% N_2_ and 5% CO_2_) for 5 min. Neonatal cardiomyocytes after transduction or EV treatment were cultured in hypoxia/hypoglycemia medium in a Napco 8000wjhypoxia incubator (1% O_2_/5% CO_2_/94% N_2_). After 9-h exposure to hypoxia/hypoglycemia, culture continued in medium with normal concentration of glucose under conventional conditions for 3h for further analysis.

### EV isolation and characterization

MSCs were cultured in Roswell Park Memorial Institute medium containing 5% EVs-depleted FBS (Systems BioSciences, Palo Alto, CA, USA) and 1% penicillin/streptomycin (Invitrogen). MSCs were seeded in a cell culture dish (100 mm) at a density of 2 × 10^5^ cells/dish and allowed to attach overnight. The medium was renewed for another 3 days of culture. When cell confluence reached approximately 80%, EVs were then detached from the conditioned medium using ExoQuick-TC EV purify reagent (Systems Biosciences, Palo Alto, CA, USA) according to the manufacturer’s instructions. The obtained EVs were resuspended in phosphate buffer saline (PBS) (approximately 200 μL) and preserved at -80°C for subsequent quantification by Protein Assay bicinchoninic acid (BCA) Kit and for molecular analysis.

Transmission electron microscopy (TEM) was used to analyze EVs structure. EVs were fixed in 2% glutaraldehyde and 2% paraformaldehyde (PFA) in 0.1 M Sorensen’s phosphate buffer for 3 h, in 1% OsO_4_ for 30 min, washed, dehydrated in graded series of 50%, 70%, 80%, 90%, and 100% ethanol and embedded in Epon 812. Ultrathin sections (60 nm) were cut with a Leica Ultracut UCT ultramicrotome, stained with uranyl acetate and Reynolds lead citrate, and observed under a TEM (JEOL JEM-1230). The size of EVs was measured using nanoparticle tracking analysis by means of NanoSight NS300 (Malvern, Amesbury, UK)

### EV internalization

As described [37], EVs (20 μg/mL) were pre-labeled by PKH67 (Sigma-Aldrich, Merck KGaA, Darmstadt, Germany) and co-cultured with neonatal cardiomyocytes in DMEM containing 10% FBS for 48h. Cold PBS wash was performed to prevent uptake of EVs by cardiomyocytes, followed by fixation in 4% PFA. EVs were observed using an Olympus BX41 microscope equipped with charge-coupled device (MagnaFire, Olympus Corp., Japan) camera. The PKH67-labeled EVs in cardiomyocytes were identified by flow cytometry after 48h culture of MSCs and cardiomyocytes at 37°C.

### Reverse transcription quantitative polymerase chain reaction (RT-qPCR)

The cardiomyocytes (5 × 10^7^) and myocardium (500 mg) were collected and detached. MSCs-derived EVs were isolated and exposed to RNase for 15 min to eliminate free-floating extra-vesicular RNA elements. The remaining RNase was removed by PBS wash and subsequent centrifugation. Total RNA and miRNA was extracted from myocardium and cardiomyocytes in the ischemic border area using Trizol (Invitrogen) and mirPremier microRNA isolation kit (Sigma, St Louis, MO, USA). In total, 10 ng total RNA from EVs were used for RT, which was conducted by First Strand cDNA kit (Thermo Fisher Scientific Inc., Rockford, IL, USA). The quantification of TXNIP and glyceraldehyde-3-phosphate dehydrogenase (GAPDH) was analyzed by LightCycler FastStart DNA Master^PLUS^ SYBR green I kit (Roche Diagnostics, Burgess Hill, UK) while that of miR-150-5p and U6 was analyzed by TaqMan microRNA analysis and TaqMan universal standard Mix II (Applied Biosystems, Foster City, CA, USA). Primer sequences are depicted in Table 2. Results were calculated by 2^−ΔΔCt^ method [38].

**Table 2:** Primer sequences for reverse transcription quantitative polymerase chain reaction.

**Gene**	**Forward (5’ - 3’)**	**Reverse (5’ - 3’)**
TXNIP	CTGAAGTTACCCGAGTCAAAGC	CTCACCTGTAGCCTGGTCTTCT
GAPDH	CTGACATGCCGCCTGGAGA	ATGTAGGCCATGAGGTCCAC
miR-150-5p	CGCCAGGGTTTTCCCAGTCACGACTCCCAACCCTTGTACCAGT	CGCGAGGAGAGAATTAATACGACTCAGTATACGCGCACTGGT
U6	CTCGCTTCGGCAGCACA	AACGCTTCACGAATTTGCGT

Note: TXNIP, thioredoxin-interacting protein; GAPDH, glyceraldehyde 3-phosphate dehydrogenase; miR, microRNA.

### Western blot analysis

Total protein was extracted from myocardium and cardiomyocytes in the ischemic border area and quantified by BCA kit (Beyotime, Jiangsu, China), followed by 10% sodium dodecyl sulfate-polyacrylamide gel electrophoresis. The protein was then transferred onto nitrocellulose membranes and the membranes was incubated with the following rabbit primary antibodies against: TXNIP (1 : 1000, ab188865, Abcam Inc., Cambridge, MA, USA), B-cell lymphoma-2 (Bcl-2) (1 : 1000, ab59348, Abcam), Bcl-2 associated protein X (Bax) (1 : 1000, #14796, Cell Signaling Technology Company, MA, USA), c-Jun (1 : 1000, #9165, Cell Signaling Technology), CD9 (1 : 2000, ab92726, Abcam), GAPDH (1 : 10000, ab108950, Abcam), CD63 (1 : 1000, ab181602, Abcam), Alix (1 : 1000, ab88743, Abcam) and glucose regulated protein 94 (GRP94) (1 : 1000, ab13509, Abcam). Then, horse radish peroxidase (HRP)-conjugated secondary antibody of goat anti-rabbit or goat anti-rat antibody (1 : 10000, ZSGB-BIO, Beijing, China) was added to incubate with the membrane. Enhanced chemiluminescence (Invitrogen) was performed for development.

### Transthoracic echocardiography

Acuson Sequoia 512 color doppler ultrasonography was conducted by professionals with the aid of a 6c2-S probe (frequency: 8.5 mHz) while the scanning speed was adjusted to 100 mm/s. Every 5 rats were selected from each group on a random basis, anesthetized by 1% pentobarbital sodium (40 mg/kg, wS20060401, Shanghai Westang Biotechnology Co., Ltd., Shanghai, China) and fixed on the testing platform, followed by the determination of the papillary muscle level M curve of the long axis and short axis of the left ventricle. Left ventricular diastolic diameter (LVEDD, mm), left ventricular systolic diameter (LVESD, mm), left ventricular end-diastolic volume (LVEDV, μL) and left ventricular end-systolic volume (LVESV, μL) were determined continuously in 3 cardiac cycles with the average calculated. Left ventricular ejection fraction (LVEEF, %) = (LVEDV - LVESV)/LVEDV × 100%, and the left ventricular fractional shortening (LVEFS, %) = (LVEDD - LVESD)/LVEDD × 100% according to the Simpson’s method. Rat cardiac function was evaluated by analyzing the above indicators.

### Triphenyltetrazolium chloride (TTC) staining

The apical tissue on the left ventricular wall was detached from 3 rats in each group and sliced into ultrathin sections (50 – 60 mm) along the long axis of the left ventricle using vibratome. The myocardium on the left ventricular wall was stained by 1% TTC (71016588, Sinopharm Chemical Reagent Co., Ltd., Shanghai, China) for observation on ultrastructure. Sections were stained by 1% tricalcium phosphate-buffered solution (2530-85-0, Guidechem, Shanghai, China). The infarcted myocardium was observed to be whitish and the surviving myocardium in normal color. The infarcted myocardium was separated from the surviving myocardium. Images were captured by Leica Digital Camera 480 (Leica Microsystems GmbH, Wetzlar, Germany). The myocardial infarct size (MIS) was determined by ImageJ 1.26 image analysis software (National Institutes of Health, Bethesda, MD, USA). MIS (mm^2^) = myocardial infarction area/left ventricular total area × 100% [36].

### Masson's staining

Myocardium located in the ischemic border area was randomly selected from 3 rats from each group and fixed in 4% PFA at 4°C for 24h. Following conventional methods of dehydration, clearing, embedding and slicing (3-μm), the sample was stained with Picric acid-Sirius red at room temperature for 30 min and then counterstained with hematoxylin (PT003, Shanghai Bogoo Biotechnology Co., Ltd., Shanghai, China) at room temperature for 2 min. Myocardium sections were observed under a polarized light microscope (XPT-480, Shanghai Zhongheng Co., Ltd., Shanghai, China), followed by Image-Pro 6 software (Media Cybernetics Inc., Bethesda, MD, USA) analysis. Five high-magnification fields were randomly selected and the myocardial collagen volume fraction (CVF) was calculated. CVF (%) = collagen area/total area × 100%, while the area surrounding the vessels did not count in the collagen area.

### Immunohistochemistry

Myocardium located in the ischemic border area was randomly selected from 3 rats in each group and fixed in 4% PFA at 4°C for 24h. The paraffin-embedded sample was cut into 3-μm serial sections by a microtome. Following the conventional steps of immunohistochemical staining, the activity of endogenous peroxidase was eliminated in 0.3% H_2_O_2_-methanol solution at room temperature for 10 min, and the sample was incubated with rabbit antibody to TXNIP antibody (ab188865, 1 : 200, Abcam) for 16h at 4°C and then with HRP-labeled goat anti-rabbit antibody to immunoglobulin G (IgG) (ab6728, Abcam) for 2h. Then, at 5 min post diaminobenzidin (DAB) (cat. no. AR1000, Wuhan Boster Biological Technology Co., Ltd., Wuhan, Hubei, China) staining, sections were observed under the microscope with images captured, which were analyzed by Image Pro Plus 6.0 software (Media Cybernetics Inc., Maryland, China). Five fields of view were selected on a random basis with the percentage of positive cells in each field obtained. The incubation with secondary antibody alone was used as NC and treatment with goat anti-rabbit antibody to IgG served as another NC. Yellow or brown staining was indicative of positive cells. The percentage of TXNIP positive cells = the number of TXNIP positive cells/the number of total cells. If the percentage was > 10%, the sample was regarded positive (+); if the percentage was ≤ 10%, it was regarded negative (-).

### Transferase deoxyuridine triphosphate-mediated 2'-deoxyuridine 5'-Triphosphate nick end labeling (TUNEL) staining

Myocardium located in the ischemic border area was selected, routinely dehydrated, fixed, embedded with paraffin and then sliced. Every 5 sections were taken from each group for dewaxing with xylene, dehydrating using a gradient ethanol and washing in PBS (pH = 7.2) for 3 times. After protease K (20 μg/mL) detachment for 30 min at 37°C, sections were washed with PBS, treated with 3% H2O2 at room temperature for 5 min to block the activity of endogenous peroxidase and washed with PBS again. The dirt accumulated on the cell membrane surface was removed by 0.1% citric acid solution with 0.1% TritonX-100 and then the sample was then frozen for 4 min on ice and rinsed with PBS. TUNEL (50 μL, batch number: ZK-8005, Zhongshan Golden Bridge Biotech Co., Ltd., Beijing, China) was added for incubation at 37°C for 60 min, followed by a PBS wash. After incubation with peroxidase at 37°C for 30 min, the sections were washed by PBS, and developed with DAB. Five non-overlapping visual fields at the ischemic border area were randomly selected and the number of TUNEL positive cells in 100 cardiomyocytes was counted with the mean value calculated. The positive rate of cell apoptosis was presented as the percentage of apoptotic cells per unit area.

Cardiomyocytes subjected to different treatments were fixed in 4% PFA for 30 min at room temperature. TUNEL was performed by means of TUNEL system (Promega, Madison, WI, USA) according to the manufacturer’s instructions, followed by a 5-min nucleus staining with 4',6-diamidino-2-phenylindole. Five fields of view were randomly selected under a fluorescence microscope (BX41, Olympus Corp., Japan), followed by a cell counting process. The number of cells with TUNEL-positive nuclei was determined. Images were captured by digital camera and analyzed by MagnaFire 2.1 software.

### Dual-luciferase reporter gene assay

The artificially synthesized TXNIP 3’untranslated region (3'UTR) was introduced into pMIR-reporter (Beijing Huayueyang Biotechnology Co., Ltd., Beijing, China) by endonuclease sites SpeI and Hind III. Mutant (MUT) sites in the complementary sequences of the seed sequence were designed based on TXNIP wild type (WT). The target fragments were inserted into pMIR-reporter by T4 DNA ligase following restriction endonuclease digestion. The correctly sequenced dual-luciferase reporters WT and MUT were co-transfected with miR-150-5p mimic into HEK-293T cells (CRL-1415, Shanghai Xinyu Biological Technology Co., Ltd., Shanghai, China), respectively. Cells were collected and lysed at 48h post transfection. The luciferase activity was determined using a luciferase detection kit (RG005, Shanghai Beyotime Biotechnology Co., Ltd., Shanghai, China) on Glomax20/20 luminometer (Promega).

### Statistical analysis

All calculations were made using SPSS 21.0 software (IBM Corp., Armonk, NY, USA). Data from *in vitro* experiments were expressed as mean ± standard error of the mean from at least three independent experiments. Data from analysis of animal models were expressed as mean ± standard deviation. Data in normal distribution and homogeneity of variance were analyzed by independent sample *t*-test between two groups. Comparison among multiple groups was analyzed by one-way analysis of variance, followed by Tukey’s post hoc test. The non-parametric test was used for data comparison that didn't coincide with normal distribution. Statistical significance was assumed when *p* < 0.05.

## Supplementary Material

Supplementary Figure 1
