# Supplementary material for: Extracellular vesicles derived from microRNA-150-5p-overexpressing mesenchymal stem cells protect rat hearts against ischemia/reperfusion
Source: Aging (Albany NY). 2020 Jul 13;12(13):12669–83. doi: 10.18632/aging.102792 (PMC7377831; doi:10.18632/aging.102792)
Supplement: Supplementary Figure 1 [file aging-12-102792-s001..pdf]

SUPPLEMENTARY FIGURE

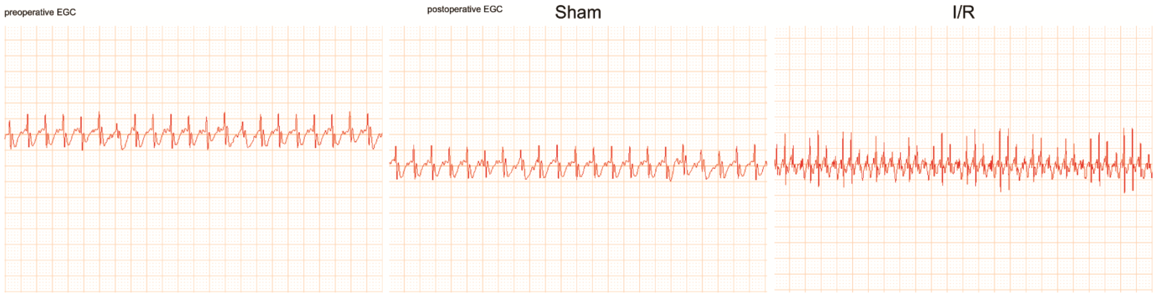

Supplementary Figure 1. Preoperative and postoperative electrocardiogram of sham-operated rats and rats with I/R.
